# The analysis of pleural complications of COVID-19 pneumonia

**DOI:** 10.3906/sag-2012-268

**Published:** 2021-04-21

**Authors:** Merve Şatır TÜRK, Irmak AKARSU, İsmail TOMBUL, Aykut KANKOÇ, Nur Dilvin ÖZKAN, Elgün VALIYEV, Muhammet SAYAN, Ali ÇELİK, İsmail Cüneyt KURUL, Olgun Kadir ARIBAŞ, Abdullah İrfan TAŞTEPE

**Affiliations:** Department of Thoracic Surgery, Faculty of Medicine, Gazi University, Ankara, Turkey

**Keywords:** Pneumothorax, pneumomediastinum, COVID-19, empyema, pandemic

## Abstract

**Background/aim:**

As the number of case reports related to the new type of coronavirus (COVID-19) increases, knowledge of and experience with the virus and its complications also increase. Pleural complications are one relevant issue. We aimed in this study to analyze pleural complications, such as pneumothorax, pneumomediastinum, and empyema, in patients hospitalized with the diagnosis of COVID-19 pneumonia.

**Materials and methods:**

The files of patients who have pleural complications of COVID-19 pneumonia and were consulted about thoracic surgery between March 2020 and December 2020 were retrospectively reviewed. The data of the patients were analyzed according to age, sex, length of stay, treatment method for pleural complications, mortality, severity of COVID-19 pneumonia, tube thoracostomy duration, and presence of a mechanical ventilator.

**Results:**

A total of 31 patients fulfilling the inclusion criteria were included in the study. There were 11 female (35.5%) and 20 male (65.5%) patients. The most common complication was pneumothorax in 20 patients (65%). The median duration of hospitalization was 22 days and the mortality rate was 71%. Mortality was significantly higher in patients on mechanical ventilation (p = 0.04).

**Conclusion:**

The mortality rate is very high in patients with pleural complications of COVID-19 pneumonia. Pneumothorax is a fatal complication in critically ill patients with COVID-19 pneumonia.

## 1. Introduction

As the number of case reports related to the new type of coronavirus (COVID-19) that emerged at the end of 2019 and caused the pandemic increases, the knowledge about its clinical conditions and complications also increases. Patients with COVID-19 pneumonia may experience a wide range of clinical conditions, from being asymptomatic to dying due to respiratory failure. COVID-19 pneumonia may be complicated by some pleural complications such as pneumothorax (PT), pneumomediastinum (PM), pleural effusion and empyema [1–4]. In the physiopathology of PM in COVID-19 pneumonia, the Macklin effect, in which alveolar ruptures form due to increased intrathoracic pressure caused by coughing, resulting in free alveolar air moving from the hilus to the mediastinum through the bronchovascular sheaths are blamed [5]. PT can occur spontaneously due to pneumonia, or it can be barotraumatic with positive airway pressure in patients on mechanical ventilators [6,7]. Empyema due to COVID-19 pneumonia is very rare in the literature and is explained by the secondary infection of the pleural effusion occurring via the inflammatory effect [8]. Here, we aimed to analyze the data of patients with pleural complications of COVID-19 pneumonia, including PT, PM, and pleural effusion, in this retrospective study.

## 2. Materials and methods

### 2.1. Patient selection

After approval by the Republic of Turkey Ministry of Health Ethics Committee (2020-10-16T13_53_24), we retrospectively reviewed the files of patients with pleural complication of COVID-19 pneumonia and who consulted with the Department of Thoracic Surgery in our hospital between March 2020 and December 2020. COVID-19 diagnoses were made via thorax computed tomography (CT), swab polymerase chain reaction (PCR) tests, or antigen-antibody tests. The patients whose follow-up records were not available, whose pleural complications were not confirmed radiologically, and whose complications occurred for iatrogenic reasons, such as central venous catheterization or thoracentesis, were not included in this study. Analyses were performed according to age, sex, length of stay, treatment methods for complications, mortality, severity of COVID-19 pneumonia, duration of tube thoracostomy and the presence of a mechanical ventilator.

### 2.2. Statistical analysis

Analyses were made using the SPSS (IBM, version 20, NY, USA) program. Descriptive data were given as mean ± standard deviation, median (minimum-maximum) or number and frequency. The chi-squared test was used for categorical variables and the log rank test was used for continuous variables. Distribution normalization was evaluated by histogram. Mean values were used for normal distribution and median values were used for asymmetrical distribution. Overall survival was investigated using the Kaplan–Meier method and survival differences between groups were investigated using the log-rank and Cox-regression methods. Studies were conducted at a 95% confidence interval; p <0.05 was considered significant.

## 3. Results

**Figure 1:**
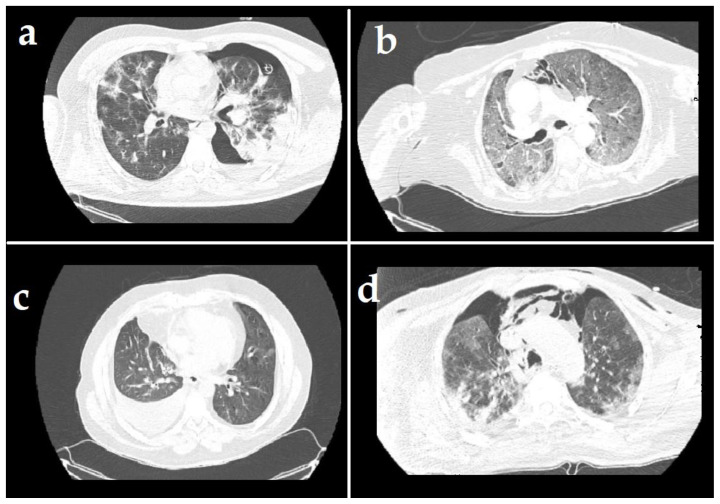
a) Thorax CT shows left-sided pneumothorax in a patient with COVID-19 pneumonia; b) Coexistence of pneumomediastinum and diffuse ground glass opacities are seen in tomography scan; c) Tomographic view of right-sided pleural effusion and mild COVID-19 pneumonia; d) Thorax CT scan shows pneumomediastinum and bilateral pneumothorax.

**Figure 2:**
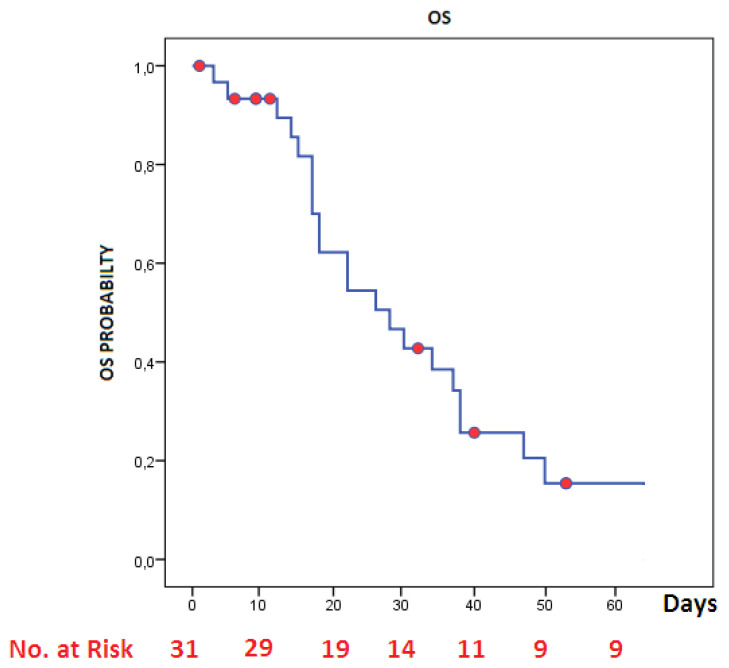
Overall survival of patients in the time of hospitalization.

**Figure 3:**
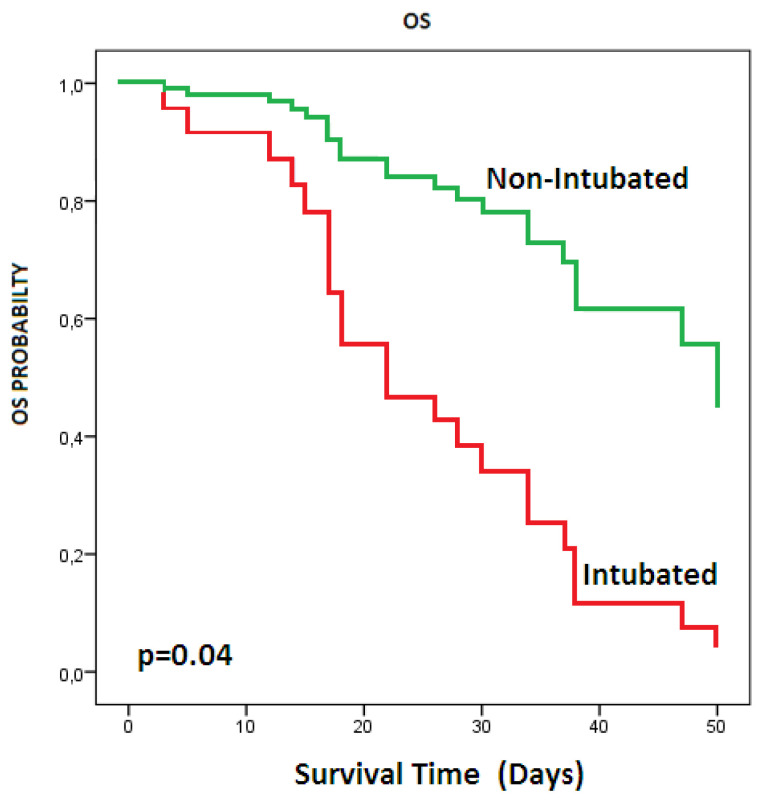
There was a statistically significant survival difference between intubated and nonintubated patients (p = 0.04).

A total of 31 patients who met inclusion criteria were included in the study. There were 11 female (35.5%) and 20 male (65.5%) patients. The characteristics of the patients are given in Table. The median age was 67 (range: 31–90). The most common pleural complication was PT in 20 patients (64.5%) followed by isolated PM in 7 patients (22.5%) and pleural effusion in 4 patients (Figures 1a–1c). Pleural complications occurred in right side in 13 patients, left side in 9 patients, and bilaterally in 2 patients (Figure 1d). The number of patients who need the mechanical ventilators was 22 (3 of them with noninvasive mechanical ventilator). Tube thoracostomy was performed in 20 patients (2 of them bilaterally). The treatment of 8 (40%) patients with pneumothorax was completed successfully and their tube thoracostomies were removed. The median hospitalization time was 22 days (range: 1–64) and the median duration of tube thoracostomy was 9 (0–38) days. A total of 22 (71%) patients died. During the 60-day follow-up, the mean survival of patients with pleural complications was 31.3 days (SD: 3.6, Figure 2). The mean survival day was significantly better in nonintubated patients than in intubated patients (43 versus 27 days, respectively, p = 0.04, Figure 3). There were no significant correlations between survival, and age (p = 0.7), etiology (p = 0.09), and being older than 70 years (p = 0.2). Sixteen of (80%) of 20 patients with pneumothorax needed mechanical ventilation. Surgical treatments such as bullae excision, primary repair, or pulmonary resection were not performed for pneumothorax in any of the patients due to their very poor general medical condition or the adequacy of tube thoracostomy treatment. The most common comorbidity of our series was hypertension in 12 (38.7%) patients, followed by malignancy in 8 (25.8%) patients. Other comorbidities were given in Table. Mortality occurred in 3 of 6 patients who had no comorbidity and 20 of 25 patients with comorbidity. The mean survival time in comorbidity-positive and -negative group were 18 and 29 days, respectively, but survival differences was not statistically significant (p = 0.1). Our series included one empyema case. The staphylococcus aureus strains were isolated in the pleural fluid culture and she was treated with tube thoracostomy, intrapleural lavage, and intravenous antibiotics for 31 days. The patient was discharged without surgical intervention.

## 4. Discussion

In this study, pleural complications occurring due to COVID-19 pneumonia were analyzed. Pleural complications occurring in COVID-19 pneumonia cases increase mortality, morbidity, interventional procedures and patient costs. PM occurs via the Macklin effect, as explained above. Due to the continuity of the mediastinal and neck fascia, free air can move to the subcutaneous area or peripheral to the subpleural region, causing PM-subcutaneous emphysema and PT, respectively [9]. Sometimes the coexistence of these three entities can be detected. In our study, there were isolated PT in 17 patients, isolated PM in 7 patients, and coexistence of PM-PT and subcutaneous emphysema in 3 patients. Mallick et al. claimed that pleural complications in COVID-19 pneumonia occurred due to parenchymal degeneration as a result of prolonged disease and severe inflammation [2]. Similarly, Oye et al. reported that the PM and PT in COVID-19 pneumonia emerged due to damaging the lung parenchyma with ischemic and inflammatory effects. They also reported that the risk of complications was increased with the severity of the disease [10]. Additionally, there are studies in the literature reporting that PT is related to positive air pressure [11,12].

Hameed et al. concluded in their series including 3 patients that tube thoracostomy was required for 2 patients and the mean duration of the tube thoracostomy was 11 days [13]. The median tube thoracostomy duration was 9 days in our study. Udi et al. indicated in their study including patients with respiratory failure requiring mechanical ventilators that barotrauma was significantly higher in the COVID-19 pneumonia group compared to other, and they explained that this was due to the excess parenchymal restriction [6]. In our study, the spontaneous PT-PM/barotraumatic PT-PM ratio was 0.25 and it supported the inference by Udi et al.. Through the literature review, we have seen that the severity of pneumonia is excessive in patients who had pleural complications of COVID-19 pneumonia [10,11,14]. In our series, 80% of the patients had severe pneumonia in accordance with the literature.

In general, there is no specific treatment for isolated PM, and treatment is based on symptoms. If severe mediastinal emphysema and subcutaneous emphysema are present, free air can be drained [15]. In our study, patients who developed isolated PM were followed up with daily chest X-ray and the mediastinal emphysema resolved in the following days spontaneously. It has been reported that the mortality of pleural complication occurring in COVID-19 pneumonia is quite high. It is not clear whether this is related to the severity of the underlying pneumonia or whether pleural complications contribute to mortality. Mortality rate in our study was over 70% and only 9 patients (29%) were discharged successfully. Cases of effusion/empyema developing after COVID-19 pneumonia are rarely reported in the literature. Tessitore et al. published a case series including 3 patients with empyema treated with surgical decortication. They reported that a strong inflammatory response and added vasculitis and microvascular thrombosis in response to infection in the lower respiratory tract caused reactive pleural effusion and empyema developed with bacterial superinfection [8]. On the other hand, Yarlagadda et al. reported that empyema occurred via aspiration pneumonia in their case report [4]. In our study, there were 3 pleural effusion and 1 empyema cases. We thought that the etiology was bacterial superinfection of the reactive fluid in our patient who had a long duration of hospitalization and had some comorbidities, such as hemodialysis for renal failure, diabetes, and hypertension. The patient was discharged without surgical intervention.

The limitations of our study were as follows; it is a retrospective and single-centered study and included small number cases. In addition, we could not make a comparison with patient groups who did not have any pleural complication of COVID-19 pneumonia or those who were hospitalized for other viral pneumonia because only patients who were consulted with our department were included in the study. Another limitation of our study was that we wanted to investigate the relationship between inflammation parameters and survival. However, these parameters were high in almost all of the patients included the study since our study group was on the hospitalized patients with COVID-19 pneumonia. In addition, we did not decide on which blood value was taken on which day of hospitalization. Additionally, the number of patients included in our study was insufficient for any analysis which determines cut-off value for inflammation markers such as receiver operating characteristics (ROC) analysis.

## 5. Conclusion

Pleural complications due to COVID-19 pneumonia can be seen rarely. Mortality is very high in patients with this complication. However, multicenter studies with more patients and long-term follow-ups are needed to clarify whether the mortality is due to complications or whether the complications develop in patients with severe pneumonia.

## Figures and Tables

**Table:** Characteristics of patients.

		n	%
Age, Med., Range: 67 years (30–90 years)			
Duration of H, Med, Range (Days): 2 (1–64)			
Duration of TT, Med, Range (Days): 9 (1–38)			
Sex			
	F	11	35.5
	M	20	65.5
Pleural complications			
	PT	17	54.8
	PM	7	22.6
	PT + PM	3	9.7
	PE	3	9.7
	Empyema	1	3.2
MV			
	Spontaneous	19	61.3
	NIMV	3	9.7
	IMV	9	29
Mortality			
	Exitus	22	71
	Alive	9	29
Treatment			
	Conservative	10	32.2
	TT	21	67.8
Side of PT, PE			
	Right	13	54.2
	Left	9	37.5
	Bilaterally	2	8.3
Comorbidities	Bronchial asthma	3	9.6
	COPD	4	12.9
	Hypertension	12	38.7
	DM	1	3.2
	Malignancy	8	25.8
	CAD	5	16.1
	CVD	3	9.6
	CRF	1	3.2
	Cirrhosis	1	3.2
	None	6	19.3

**Abbreviations**: CAD: coronary artery disease; COPD: chronic obstructive pulmonary disease; CRF: chronic renal failure; CVD: cerebro-vascular disease; DM: diabetes mellitus; F: female, IMV: invasive mechanical ventilation, H: hospitalization, M: male; Med: median; MV: mechanical ventilation; NIMV: noninvasive mechanical ventilation; PE: pleural effusion;

PM: pneumomediastinum; PT: pneumothorax, TT: tube thoracostomy.
